# Complement Alternative and Mannose-Binding Lectin Pathway Activation Is Associated With COVID-19 Mortality

**DOI:** 10.3389/fimmu.2021.742446

**Published:** 2021-09-10

**Authors:** Federica Defendi, Corentin Leroy, Olivier Epaulard, Giovanna Clavarino, Antoine Vilotitch, Marion Le Marechal, Marie-Christine Jacob, Tatiana Raskovalova, Martine Pernollet, Audrey Le Gouellec, Jean-Luc Bosson, Pascal Poignard, Matthieu Roustit, Nicole Thielens, Chantal Dumestre-Pérard, Jean-Yves Cesbron

**Affiliations:** ^1^Laboratoire d’Immunologie, Institut de Biologie et Pathologie, Centre Hospitalier Universitaire Grenoble Alpes, Grenoble, France; ^2^Cellule d’Ingénierie des Données, Centre Hospitalier Universitaire Grenoble Alpes, Grenoble, France; ^3^Centre d’Investigation Clinique de l’Innovation et de la Technologie (CIC-IT), Centre Hospitalier Universitaire Grenoble Alpes, Grenoble, France; ^4^Service des Maladies Infectieuses et Tropicales, Centre Hospitalier Universitaire Grenoble Alpes, Grenoble, France; ^5^Université Grenoble Alpes, TIMC-IMAG, Grenoble, France; ^6^Laboratoire de Biochimie, Institut de Biologie et Pathologie, Centre Hospitalier Universitaire Grenoble Alpes, Grenoble, France; ^7^Université Grenoble Alpes, CNRS, CEA, Institut de Biologie Structurale (IBS), Grenoble, France; ^8^Laboratoire de Virologie, Institut de Biologie et Pathologie, Centre Hospitalier Universitaire Grenoble Alpes, Grenoble, France; ^9^Département de Pharmacologie Clinique INSERM CIC 1406, Centre Hospitalier Universitaire Grenoble Alpes, Grenoble, France; ^10^Université Grenoble Alpes, UMR 1042-HP2, INSERM, Grenoble, France

**Keywords:** COVID-19, complement, alternative pathway, MBL, lectin pathway

## Abstract

**Background:**

The SARS-CoV-2 infection triggers excessive immune response resulting in increased levels of pro-inflammatory cytokines, endothelial injury, and intravascular coagulopathy. The complement system (CS) activation participates to this hyperinflammatory response. However, it is still unclear which activation pathways (classical, alternative, or lectin pathway) pilots the effector mechanisms that contribute to critical illness. To better understand the immune correlates of disease severity, we performed an analysis of CS activation pathways and components in samples collected from COVID-19 patients hospitalized in Grenoble Alpes University Hospital between 1 and 30 April 2020 and of their relationship with the clinical outcomes.

**Methods:**

We conducted a retrospective, single-center study cohort in 74 hospitalized patients with RT-PCR-proven COVID-19. The functional activities of classical, alternative, and mannose-binding lectin (MBL) pathways and the antigenic levels of the individual components C1q, C4, C3, C5, Factor B, and MBL were measured in patients’ samples during hospital admission. Hierarchical clustering with the Ward method was performed in order to identify clusters of patients with similar characteristics of complement markers. Age was included in the model. Then, the clusters were compared with the patient clinical features: rate of intensive care unit (ICU) admission, corticoid treatment, oxygen requirement, and mortality.

**Results:**

Four clusters were identified according to complement parameters. Among them, two clusters revealed remarkable profiles: in one cluster (n = 15), patients exhibited activation of alternative and lectin pathways and low antigenic levels of MBL, C4, C3, Factor B, and C5 compared to all the other clusters; this cluster had the higher proportion of patients who died (27%) and required oxygen support (80%) or ICU care (53%). In contrast, the second cluster (n = 19) presented inflammatory profile with high classical pathway activity and antigenic levels of complement components; a low proportion of patients required ICU care (26%) and no patient died in this group.

**Conclusion:**

These findings argue in favor of prominent activation of the alternative and MBL complement pathways in severe COVID-19, but the spectrum of complement involvement seems to be heterogeneous requiring larger studies.

## Introduction

The severe acute respiratory syndrome coronavirus 2 (SARS-CoV-2) infection drives sustained inflammatory response considered to be a major cause of disease severity and death in patients with COVID-19 (1). Growing evidence suggests that the complement system (CS) activation instigates this dysregulated inflammatory reaction in COVID-19: elevated levels of the anaphylatoxin C5a have been reported to be proportional to disease severity (2); the membrane attack complex concentration has been linked with respiratory failure and systemic inflammation in infected patients (3); and deposits of mannose-binding lectin (MBL) and MBL-associated protease MASP-2 have been found in the microvasculature of critical patients with SARS CoV-2 infection (4). On the other hand, patients treated with complement blockers (anti-C5a mAb [eculizumab] or C3-inhibitor) exhibited a drop in inflammatory markers and significant clinical improvement (5–9). However, it is still not fully understood which of the three complement activation pathways (classical, alternative, or lectin pathway) drives the effector mechanisms that contribute to the tissue injury. To address these questions, we performed extensive analysis of CS activation pathways and components in samples collected from hospitalized COVID-19 patients and their relationship with the clinical outcomes.

## Methods

### Study Participants

This retrospective, single center study included 74 patients with RT-PCR-proven COVID-19 admitted to Grenoble Alpes University Hospital from April 1 to 30, 2020. Samples were collected during hospitalization in infectious/pneumology/internal medicine department or intensive care unit (ICU) of our hospital. The study was performed in accordance with the Declaration of Helsinki, Good Clinical Practice guidelines, and CNIL (Commission Nationale de l’Informatique et des Libertés) methodology reference. Patients were informed and consent was obtained, according to French law. Demographic, clinical characteristics (oxygen requirements, ICU admission, mortality, steroid treatment) and laboratory data were collected from electronic clinical records and included in an anonymized database. Patients were classified as severe on the basis of oxygen requirement (>2 L O_2_/min), ICU admission, limitation of therapeutic effort (LTE), and mortality, according to (10).

### Complement Testing

Peripheral blood samples were collected in citrate anticoagulated or without anticoagulant tubes for hemolytic and functional assays or antigenic level measurement, respectively. Total hemolytic assays for classical pathway (CP, TH50c) and alternative pathway (AP, TH50a) were assessed as previously published (11); 100% lysis is defined by the TH50c/TH50a of the control sample. Reference values (TH50c: 86–126%; TH50a: 84–150%) were established by testing samples from 50 blood donors.

Antigenic levels of C1q, C4, C3, C5, and Factor B proteins in the serum samples were measured using a laser nephelometer BNII (Dade Behring, GmbH, Marburg, Germany). Reference intervals (RI; C1q: 154–258 mg/L; C4: 100–380 mg/L; C3: 880–1650 mg/L; C5: 120–220 mg/L; Factor B: 216–504 mg/L) were obtained by testing samples from 50 blood donors.

Determination of MBL protein concentration and function was realized by an enzyme-linked immunosorbent assay (ELISA) as described previously (12). The characterization of MBL protein expression deficiency was established by the combination of three assays: ELISA for antigen and functional MBL and hemolytic activity of C4 (C4H) (13) normal values of C1q confirmed the absence of CP activation. Low values of antigenic (<100 µg/L) (14) and functional MBL associated with a normal value of C4H defined patients with MBL protein expression deficiency. Low levels of antigenic and functional MBL associated with decreased value of C4H identified patients with MBL pathway activation. Reference values for MBL protein concentration and function and C4H were determined from 50 blood donors (MBL antigen: 30–3000 µg/L; MBL function: 35–115%; C4H: 70–130%).

### Statistical Analysis

Statistical analysis was performed using hierarchical ascendant clustering (HAC) in order to identify groups of COVID-19 patients with similar characteristics (“clusters”) in terms of complement variables: TH50c, TH50a, C1q, C4, C3, C5, Factor B, and MBL antigen. Age was included in the model (**Supplementary Methods**).

The biological significance of the clusters was analyzed by comparing the values of every complement parameter between the clusters. The ANOVA F-test was performed for variables with a Gaussian distribution, and the Kruskal-Wallis test for the variables with other distribution. Mean (standard deviation [sd]) or median (interquartile range [IQR]) were presented for the parametric or non-parametric variables, respectively. For the markers with a significant difference between the clusters, specific cluster by cluster tests were performed using Student or Wilcoxon tests to identify the cluster significantly different from the others. Post-hoc analysis with the Fisher’s exact test was used to test specific difference between the clusters.

## Results

**Table 1** details the main demographic and clinical characteristics of our cohort. The median age of patients was 72 years (IQR: 62;82; range: 32–96); more than half of patients were men (58%). Of the 74 patients, 43 (58%) were severe, 8 (11%) died, 23 (31%) were admitted in the ICU, 3 (4%) were with LTE, and 23 (31%) required treatment by corticoids.

**Table 1:** Patient characteristics.

Clinical and biological characteristics	
n	74
Age: median (IQR) (min-max), years	72 (62;82) (32–96)
Sex: men/women	43/31
CRP: mean (sd), mg/L	71 (66.4)
CRP: median (IQR), mg/L	59 (22-92)
***Prognosis/outcome, n (%)***	
Severe COVID-19^1^, n (%)	43 (58)
Mortality	8 (11)
ICU admission	23 (31)
Limitation of therapeutic effort, n (%)	3 (4)
Corticosteroid treatment, n (%)	23 (21)
***Oxygen support:***	
>2 L O_2_/min	39 (53)
≤2 L O_2_/min	13 (18)

^1^Severe COVID-19 defined as: O_2_ > 2 L/min, ICU [intensive care unit] admission, LTE [limitation of the therapeutic effort], decease.

The results of complement proteins and activation pathways analysis performed in samples collected during hospitalization are summarized in **Table 2**.

**Table 2:** Complement parameters.

	RI	Mean	sd	Min	Lower quart	Median	Upper quart	Max
**TH50c (%)**	86–126	124	30	67	101	121	142	234
**TH50a (%)**	84–150	122	70	23	71	102	155	317
**C1q (mg/L)**	154–258	208	48	71	195	212	234	336
**C4 (mg/L)**	100–380	318	114	57	249	327	402	551
**C3 (mg/L)**	880–1650	1339	357	603	1050	1355	1550	2210
**Factor B (mg/L)**	216–504	466	148	182	345	465	581	828
**C5 (mg/L)**	120–220	225	52	142	186	214	260	367
**MBL antigen (µg/L)**	30–3000	1344	1586	20	270	775	1750	9000
**MBL function (%)**	35–115	94	79	0	22	66	178	200
**C4 hemolytic activity (%)**	70–130	106	51	5	76	100	132	251

RI, reference interval.

**Figure 1:**
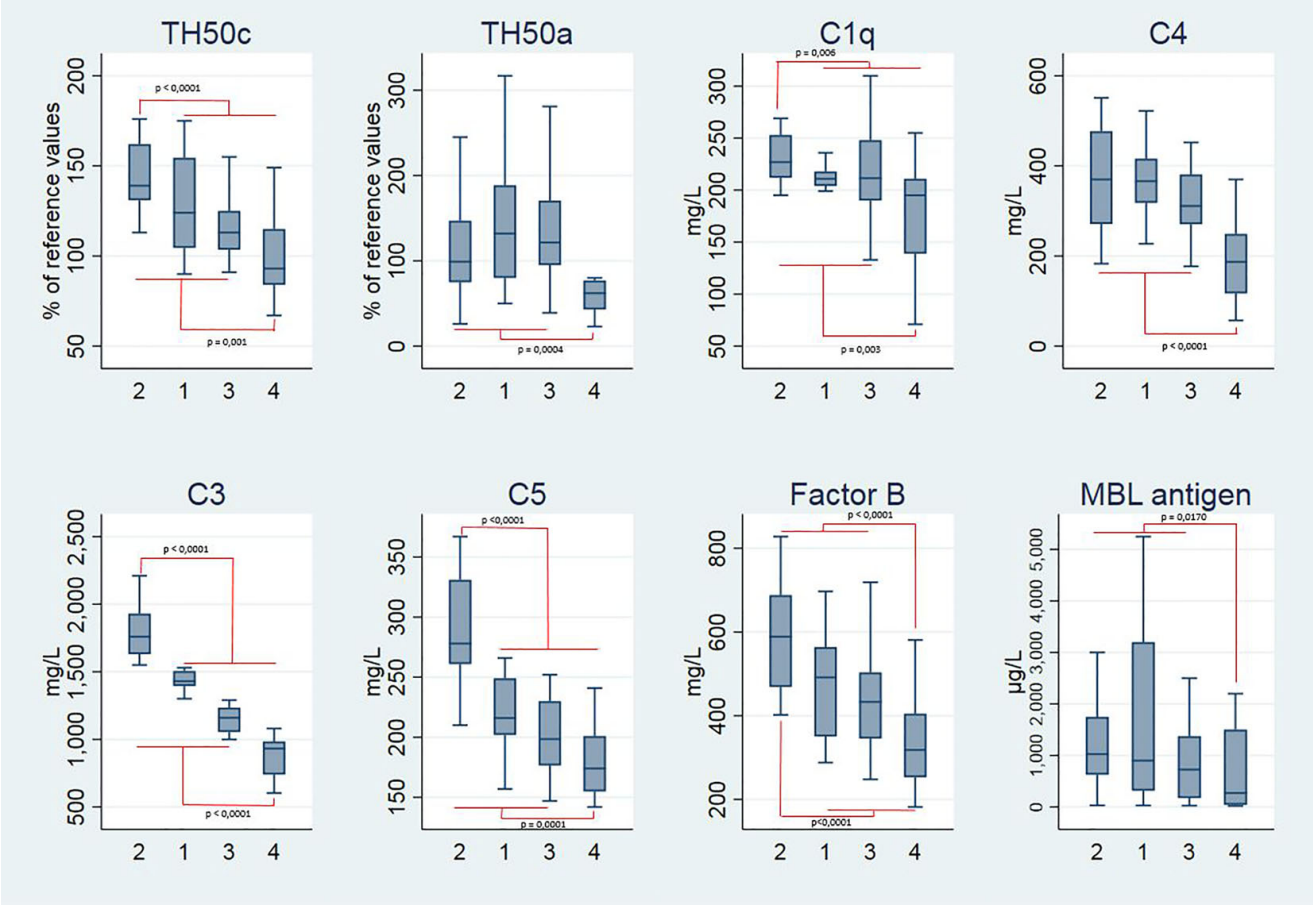
Boxplots representing the complement parameters of the four clusters of patients. Statistical analysis by hierarchical ascendant clustering discriminates the 74 patients with COVID-19 of the cohort in four distinct clusters according to complement variables: TH50c, TH50a, C1q, C4, C3, C5, Factor B, and MBL antigen. Age was included in the model. Boxplots represent the median and 25^th^ to 75^th^ percentiles, the whiskers denote the maximum and minimum values, and the horizontal bars indicate the medians. Outside values were excluded.

Four clusters of individuals were identified in the studied cohort by HAC analysis according to the complement data (**Supplementary Figure S1**). The comparison between the clusters (cluster 2 against others and/or cluster 4 against others) is presented in **Figure 1** and **Table 3**. There was no statistically significant difference among the four clusters for sex, age, CRP, severity, ICU admission, O_2_ requirement, or corticoid treatment (**Table 4**).

**Table 3:** Clusters of COVID-19 patients according to complement parameters.

	RI	Cluster 1	Cluster 2	Cluster 3	Cluster 4	p-value	p-value comparison 2 by 2
Number		20	19	20	15		
**TH50c** % mean (sd)	86–126	128.1 (27.1)	145.2 (27.3)	117.5 (22.7)	100.6 (24.8)	<0.0001^1^	p = 0.001 cluster 4 against others; p < 0.0001 cluster 2 against others
**TH50a** % median (IQR)	84–150	132 (80;189)	99 (75;147)	122 (95;171)	62 (43;77)	0.003^2^	p = 0.0004 cluster 4 against others
**C1q** mg/L mean (sd)	154–258	201.4 (40.1)	234.3 (32.5)	215.9 (52.2)	175.2 (52.4)	0.003^1^	p = 0.003 cluster 4 against others: p = 0.006 cluster 2 against others
**C4** mg/L mean (sd)	100–380	367.8 (77.8)	374.7 (112.9)	317.1 (79.5)	182.3 (85.1)	<0.0001^1^	p < 0.0001 cluster 4 against others
**C3** mg/L mean (sd)	880–1650	1440 (66.7)	1795.3 (189.8)	1149.5 (99.0)	878.1 (149.9)	<0.0001^1^	p < 0.0001cluster 2 against others; cluster 4 against others
**Factor B** mg/L mean (sd)	216–504	473.5 (134.0)	585.6 (122.6)	436.9 (110.5)	345 (134.8)	<0.0001^1^	p < 0.0001 cluster 2 against others; cluster 4 against others
**C5** mg/L median (IQR)	120–220	216 (202;249)	278 (261;331)	199 (177;230)	174 (155;201)	<0.0001^2^	p < 0.0001 cluster 2 against others; p = 0.0001 cluster 4 against others
**MBL antigen** µg/L median (IQR)	30–3000	900 (318;3200)	1025 (625;1750)	725 (171;1375)	270 (42;1500)	0.0374^2^	p = 0.0170 cluster 4 against others
MBL function* % median (IQR)	35–115	110 (24;187)	117 (62;200)	43 (28;181)	13 (0;168)	0.0558^2^	
C4 hemolytic activity* % mean (sd)	70–130	125 (8.1)	117.3 (50.8)	104.1 (40.8)	66.6 (49.0)	0.004^1^	p = 0.001 cluster 4 against others

Mean (sd) or median (IQR) were presented for the parametric or non-parametric variables, respectively. RI, reference interval.

*Post-hoc analysis was performed for this parameter. ^1^ Fisher ANOVA test. ^2^ Kruskal-Wallis test.

**Table 4:** Overall comparison of clinical and biological characteristics between the clusters.

	Cluster 1	Cluster 2	Cluster 3	Cluster 4	p-value global test
Number	20	19	20	15	
Men, n (%)	12 (60%)	14 (74%)	8 (40%)	9 (60%)	0.2109^1^
Age, (years) mean, sd	70 (15)	69 (14)	72 (14)	73 (14)	0.6265^2^
CRP, (mg/L) mean (sd) (n=73)	89.4 (84.89)	82.1 (70.77)	47.8 (44.08)	63.7 (53.86)	0.2598^2^
Severe COVID-19^3^, n (%)	14 (70%)	9 (47.37%)	9 (45%)	11 (73.33%)	0.1912^1^
ICU admission, n (%)	6 (30%)	5 (26.32%)	4 (20%)	8 (53.33%)	0.199^1^
Oxygen requirement, n (%)	18 (90%)	13 (68.42%)	11 (55%)	12 (80%)	0.774^1^
Mortality, dead, n (%)	2 (10%)	0 (0%)	2 (10%)	4 (26.67%)	0.048^4^
Corticoid requirement, n (%)	10 (50%)	4 (21.05%)	2 (10%)	7 (46.67%)	0.0181^1^

Reference values: CRP < 10 mg/L.

^1^Fisher exact test. ^2^ Kruskal-Wallis test. ^3^ Severe COVID-19 defined as: O_2_ > 2 L/min, ICU [intensive care unit] admission, LTE [limitation of the therapeutic effort], decease. ^4^ Post-hoc analysis with Fisher exact test.

For patients of the first cluster (n = 20; 27%), the complement profile was overall without anomalies (TH50c: 128%; TH50a: 132%; C1q: 201 mg/L; C4: 368 mg/L; C3: 1440 mg/L; C5: 216 mg/L; Factor B: 474 mg/L; MBL antigen: 900 µg/L; MBL function: 110%; C4H: 125%; **Table 3**). A large percentage of patients in this cluster was severe (n = 14; 70%) and required corticoid treatment (n = 10; 50%) (**Table 4**).

Patients of cluster 2 (n = 19; 26%) exhibited an inflammatory profile: high values of CP, LP, and C4 activities (TH50c: 145%; MBL function: 117%; C4H: 118%) and increased antigenic levels of C3 (1795 mg/L), C5 (278 mg/L), Factor B (586 mg/L), and MBL (1025 µg/L) were observed in this cluster compared to other clusters (**Table 3**). The majority of patients of the second cluster were men (men/women: 14/5). Interestingly, none of the patients died in this cluster (**Table 4**).

In cluster 3 (n = 20; 27%), most complement markers were within reference interval (TH50c: 118%; TH50a: 122%; C1q: 216 mg/L; C4: 317 mg/L; C3: 1150 mg/L; C5: 199 mg/L; Factor B: 437 mg/L; MBL antigen: 725 µg/L; C4H: 104%); only MBL function was decreased (43%) (**Table 3**). The third cluster was characterized by the lowest rate of ICU admission (20%) and by the lowest rate of patients having required corticoid treatment (10%); the rate of mortality was also low in this cluster (10%) (**Table 4**).

Finally, the fourth cluster (n = 15; 20%) (**Tables 3**, **4**; **Supplementary Table S1**) was specifically characterized by significant activation of alternative (TH50a: 62%; p = 0.0004 against other clusters) and lectin (MBL antigen: 270 µg/L: p = 0.0170 against other clusters) complement pathways. Decreased MBL concentration was associated with reduced MBL function (13%) and C4H (67%) confirming the MBL pathway activation. TH50c was also decreased compared to the other clusters (101%; p = 0.001), but remained in the RI (86–126%). Interestingly, in this cluster, 13 patients (87%) exhibited AP activation and 7 patients (47%) exhibited MBL pathway activation; 6 (40%) patients presented simultaneously AP and MBL pathway activation (**Supplementary Table S1**). A large percentage of patients of the fourth cluster was severe (73%) and required ICU admission (53%) and corticoid treatment (47%) (**Table 4**). Among the 11 severe patients, 10 exhibited AP activation; 6 exhibited MBL pathway activation, and 5 presented at the same time AP and MBL pathway activation (**Supplementary Table S1**). Of note, cluster 4 was characterized by a higher mortality rate (27% versus 10%, 0% and 10% in clusters 1, 2, and 3, respectively; p = 0.048) (**Table 4**). Among the four patients who died, three presented AP activation, two exhibited MBL pathway activation, and two patients showed concurrent activation of alternative and MBL pathways (**Supplementary Table S1**). Regarding the four other patients who died, all presented a normal complement profile except patient 1 who exhibited an MBL deficiency (**Supplementary Table S2**).

## Discussion

SARS-CoV-2 infection triggers an innate immune response including CS activation which is a key weapon both implicated in disease resolution and organ damage depending on the time of infection (15).

Recent studies addressing the role of complement in the pathogenesis of COVID-19 have shown a relationship between respiratory failure, intravascular coagulopathy, and complement overactivation in COVID-19 patients (3, 16). There are several lines of evidence for local deposition of complement proteins and activation products in lung, skin, and other tissues showing activation of the three pathways, CP, LP, and AP (17–21). Furthermore, systemic complement activation and consumption were related to severe COVID-19 and predictive of in-hospital mortality (22). However, despite *in vitro* lines of evidence suggesting that the SARS-CoV-2 spike proteins activate the AP (23), it remains incompletely understood which complement activation pathways contribute to critical illness in COVID-19.

Concerning the possible involvement of MBL in coronavirus infection, it has been described so far for SARS-CoV in several studies. Among those, two *in vitro* studies demonstrated binding of MBL to SARS-CoV or viral particles pseudotyped with SARS-CoV spike protein and activation of the lectin complement pathway (24, 25). More recently, Ali and *al* showed binding of LP recognition molecules to S- and N- proteins of SARS-CoV-2, as also robust LP activation on the surface of HEK 293 cells expressing SARS-CoV-2 S protein (26).

One of the limitation of the study is that it reports data about the original variant of the virus, while the delta variant is now a majority among the infected patients. The crucial mutations leading to delta variant concern the S1 subunit (intimately involved in the initiation of infection) for which we have no data concerning its molecular interaction with complement components. However, the replication rate of delta strain is much higher. It would therefore be interesting to compare our results with samples collected from patients infected with the delta variant of the virus.

Using an original, unsupervised statistical approach by hierarchical clustering on complement and clinical parameters, this study reveals for the first time the association between AP and LP activation, and the mortality in COVID-19 patients (cluster 4). Our data are in line with Ma et al. showing increased AP components in COVID-19 patients with worse prognosis (27) and with Sinkovits et al. relating significant association between AP activity and COVID-19 severity (22).

Furthermore, our results provide additional evidence for an association between MBL pathway activation and mortality, supported by the data of Eriksson et al. (4). In contrast, Sinkovits et al. reported that the LP activity showed no difference between severity groups (22). Our data are consistent with recent reports describing deposits of MBL and MASP2 in affected tissues of COVID-19 patients and *in vitro* MBL pathway activation by recombinant SARS-CoV2 proteins (17, 28). Collectively, these findings are consistent with the CS implication in the pathogenesis of severe COVID-19 infection. As consequences of unrestrained complement activation, the strong pro-inflammatory C5a-C5aR axis promotes neutrophil/monocyte infiltration and the “cytokine storm” driving lung inflammation and injury, responsible for complications in hospitalized COVID-19 patients (2).

If complement activation is evident from our results and associated with the severity of the disease, the spectrum of involvement of the complement cascade in COVID-19 seems to be heterogeneous and depending on patients: notably, complement activation could be deleterious in some ones and expression of the severity of the disease in other ones. A recent review summarizes the current knowledge about modulating complement cascade as therapeutic approach in COVID-19 patients (29). In brief, despite nonconclusive studies, the available data suggested favorable outcomes in a small number of patients with severe COVID-19 treated either with C1 inhibitor, MASP-2 monoclonal antibodies, compstatin-based complement C3 inhibitor, anti-C5 drugs, or C5a-C5aR1 antagonists. Clinical trials of complement inhibitors in COVID-19 are ongoing (NCT04288713, NCT04414631, NCT04395456). However, awaiting final results from the clinical trials, the potential benefits from complement inhibition in COVID-19 remain to be elucidated.

Interestingly, we found high incidence of MBL expression deficiency in patients of our cohort (16%, established as described in Methods), all clusters combined. Further studies based on MBL genotyping would be of interest to support biochemical data. MBL deficiency is fairly common, affecting approximately 5–10% of individuals and usually associated with increased susceptibility to bacterial infections of the upper respiratory in young children (30).

Our results highlight the dichotomous nature of the complement MBL pathway: on one hand, MBL appears to contribute to the pathogenesis of disease because it mediates complement activation that is related to clinical deterioration of patients; on the other hand, MBL could have a protective role against SARS-CoV-2 infection by promoting phagocytosis and virus lysis or neutralization. Further *in vitro* studies using pseudoviral particles or the SARS-CoV-2 virus are necessary to check the latter hypothesis.

In summary, our study suggests that alternative and lectin pathways assessment might be useful as biomarker of disease severity. Extensive investigations of complement pathways have to be performed on a larger cohort of patients with SARS-CoV-2 infection to help rationalize therapeutic choices.

## Data Availability Statement

The original contributions presented in the study are included in the article/**Supplementary Material**. Further inquiries can be directed to the corresponding author.

## Ethics Statement

The studies involving human participants were reviewed and approved by Commission Nationale de l'Informatique et des Libertés (CNIL). Written informed consent for participation was not required for this study in accordance with the national legislation and the institutional requirements.

## Author Contributions

Study conception: FD, CD-P, and J-YC. Immunological determinations: FD, CD-P, M-CJ, TR, and MP. Methodology: MR. Statistical analysis: CL, AV, and J-LB. Collection of patients’ samples and clinical information: OE and MM. Funding acquisition: FD, CD-P, NT, OE, GC, AG, and PP. Writing original draft: FD and CD-P. Review manuscript: J-YC and NT. All authors contributed to the article and approved the submitted version.

## Funding

This work was supported by funding from the Université Grenoble Alpes (projects COMPLEC-COV and BIOMARCOVID).

## Conflict of Interest

The authors declare that the research was conducted in the absence of any commercial or financial relationships that could be construed as a potential conflict of interest.

## Publisher’s Note

All claims expressed in this article are solely those of the authors and do not necessarily represent those of their affiliated organizations, or those of the publisher, the editors and the reviewers. Any product that may be evaluated in this article, or claim that may be made by its manufacturer, is not guaranteed or endorsed by the publisher.

## Abbreviations

AP, alternative pathway; COVID-19, coronavirus disease 19; CP, classical pathway; CS, Complement system; C4H, C4 hemolytic activity; LTE, limitation of the therapeutic effort; LP, lectin pathway; MBL, mannose-binding lectin; RI, reference interval.
