# Supplementary material for: Complement Alternative and Mannose-Binding Lectin Pathway Activation Is Associated With COVID-19 Mortality
Source: Front Immunol. 2021 Sep 10;12:742446. doi: 10.3389/fimmu.2021.742446 (PMC8461024; doi:10.3389/fimmu.2021.742446)
Supplement: Supplementary file 1 [file DataSheet_1.docx]

**Statistical analysis**

The data were analyzed using a non-supervised analysis: hierarchical ascendant clustering (HAC). The goal of this method is to cluster the entire analyzed population. This use an iterative way to group observations in clusters, by a distance and an aggregation method. The end of iterations results in a unique cluster made up of the whole observations. We can illustrate results with a dendrogram (Supplementary Figure 1). HAC need two parameters a priori:

- A distance: in this study the Euclidian distance

- An aggregation method: in this study the Ward’s linkage method

HAC results in all the possible clusters with the previously parameters, and defined variables. In our case, variables are the following: TH50c, TH50a, C1q, C4, C3, Factor B, C5, MBL antigen. Age was included in the model.

HAC was implemented using Stata 15 (StataCorp, College Station, TX, USA), with the command cluster ‘wardslinkage’. The optimal number of clusters was chosen using selection criterions of Calinski-Harabasz and Duda-Hart, with the following command: ‘cluster stop’.

**Supplementary Figure 1: Clustering of the patients.** The hierarchical ascendant clustering (HAC) method identified 4 groups or clusters of individuals in our cohort according to the complement data.
