# Supplementary figures and images for: Complement Alternative and Mannose-Binding Lectin Pathway Activation Is Associated With COVID-19 Mortality
Source: Front Immunol. 2021 Sep 10;12:742446. doi: 10.3389/fimmu.2021.742446 (PMC8461024; doi:10.3389/fimmu.2021.742446)

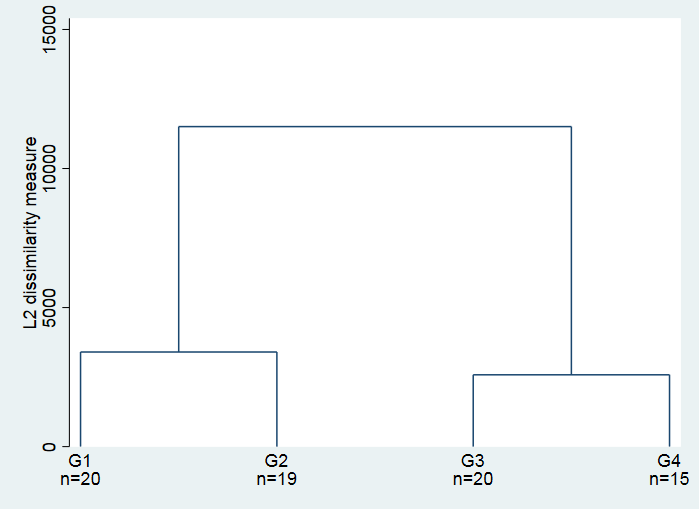

Supplement: Supplementary file 2 [file Image_1.png]
