# Supplementary material for: Complement Alternative and Mannose-Binding Lectin Pathway Activation Is Associated With COVID-19 Mortality
Source: Front Immunol. 2021 Sep 10;12:742446. doi: 10.3389/fimmu.2021.742446 (PMC8461024; doi:10.3389/fimmu.2021.742446)
Supplement: Supplementary file 3 [file Table_1.docx]

**Supplementary Table 1. Clinical and biological characteristics of the 15 patients of the cluster 4.** RI, reference interval. C4H, C4 hemolytic activity.

|  | **TH50c %** | **TH50a %** | **C1q mg/L** | **C4 mg/L** | **C3 mg/L** | **Factor B mg/L** | **C5 mg/L** | **MBL antigen µg/L** | **MBL function %** | **C4H %** | **Outcome** | **Severity class^1^** |
| --- | --- | --- | --- | --- | --- | --- | --- | --- | --- | --- | --- | --- |
| *RI* | *86-156%* | *84-150%* | *154-258* | *100-380* | *880-1650* | *216-504* | *120-220* | *30-3000* | *35-115* | *70-130* |  |  |
| Patient 1 | 69 | 207 | 232 | 263 | 614 | 278 | 143 | 662 | 51 | 132 | dead | severe |
| Patient 2 | 56 | 188 | 117 | 957 | 957 | 271 | 208 | 2200 | 168 | 16 | not dead | severe |
| Patient 3 | 111 | 48 | 197 | 370 | 932 | 449 | 174 | 83 | 0 | 120 | dead | severe |
| Patient 4 | 108 | 77 | 195 | 82 | 977 | 384 | 152 | 30 | 0 | 28 | not dead | severe |
| Patient 5 | 134 | 51 | 71 | 204 | 693 | 655 | 241 | 2000 | 174 | 158 | not dead | mild |
| Patient 6 | 88 | 43 | 211 | 187 | 1080 | 182 | 201 | 1500 | 178 | 44 | not dead | severe |
| Patient 7 | 89 | 62 | 139 | 137 | 988 | 195 | 184 | 20 | 0 | 40 | not dead | severe |
| Patient 8 | 149 | 71 | 255 | 222 | 848 | 405 | 142 | 270 | 13 | 63 | dead | severe |
| Patient 9 | 115 | 70 | 233 | 163 | 863 | 247 | 155 | 74 | 0 | 5 | not dead | mild |
| Patient 10 | 84 | 42 | 149 | 127 | 739 | 253 | 169 | 42 | 0 | 57 | not dead | severe |
| Patient 11 | 138 | 68 | 95 | 195 | 603 | 581 | 189 | 1500 | 200 | 120 | not dead | mild |
| Patient 12 | 67 | 301 | 195 | 57 | 985 | 318 | 187 | 90 | 11 | 9 | not dead | severe |
| Patient 13 | 82 | 23 | 196 | 249 | 916 | 253 | 217 | 300 | 52 | 79 | not dead | mild |
| Patient 14 | 93 | 28 | 131 | 87 | 1040 | 345 | 174 | 625 | 24 | 28 | dead | severe |
| Patient 15 | 97 | 80 | 141 | 274 | 936 | 359 | 171 | 40 | 11 | 100 | not dead | severe |

^1^ Severe COVID-19 defined as: O_2_>2L/min, ICU [intensive care unit] admission, LTE [limitation of therapeutic effort], decease
