# Supplementary material for: Complement Alternative and Mannose-Binding Lectin Pathway Activation Is Associated With COVID-19 Mortality
Source: Front Immunol. 2021 Sep 10;12:742446. doi: 10.3389/fimmu.2021.742446 (PMC8461024; doi:10.3389/fimmu.2021.742446)
Supplement: Supplementary file 4 [file Table_2.docx]

**Supplementary Table 2. Biological characteristics of the 8 patients died.** RI, reference interval. C4H, C4 hemolytic activity.

|  | **TH50c %** | **TH50a %** | **C1q mg/L** | **C4 mg/L** | **C3 mg/L** | **Factor B mg/L** | **C5 mg/L** | **MBL antigen µg/L** | **MBL function %** | **C4H %** | **Severity class^1^** | **Cluster** |
| --- | --- | --- | --- | --- | --- | --- | --- | --- | --- | --- | --- | --- |
| *RI* | *86-156%* | *84-150%* | *154-258* | *100-380* | *880-1650* | *216-504* | *120-220* | *30-3000* | *35-115* | *70-130* |  |  |
| Patient 1 | 100 | 93 | 213 | 478 | 1380 | 292 | 205 | 355 | 22 | 251 | severe | 1 |
| Patient 2 | 131 | 249 | 211 | 337 | 1500 | 531 | 173 | 3500 | 200 | 166 | severe | 1 |
| Patient 3 | 164 | 129 | 144 | 274 | 1000 | 719 | 252 | 3500 | 174 | 158 | severe | 3 |
| Patient 4 | 118 | 151 | 310 | 277 | 1180 | 337 | 186 | 1250 | 150 | 100 | severe | 3 |
| Patient 5 | 69 | 207 | 232 | 263 | 614 | 278 | 143 | 662 | 51 | 132 | severe | 4 |
| Patient 6 | 111 | 48 | 197 | 370 | 932 | 449 | 174 | 83 | 0 | 120 | severe | 4 |
| Patient 7 | 149 | 71 | 255 | 222 | 848 | 405 | 142 | 270 | 13 | 63 | severe | 4 |
| Patient 8 | 93 | 28 | 131 | 87 | 1040 | 345 | 174 | 625 | 24 | 28 | severe | 4 |

^1^ Severe COVID-19 defined as: O_2_>2L/min, ICU [intensive care unit] admission, LTE [limitation of therapeutic effort], decease
